# Metabarcoding using nanopore sequencing enables identification of diverse and zoonotic vector-borne pathogens from neglected regions: A case study investigating dogs from Bhutan

**DOI:** 10.1016/j.onehlt.2024.100839

**Published:** 2024-06-14

**Authors:** Lucas G. Huggins, Ugyen Namgyel, Pelden Wangchuk, Ushani Atapattu, Rebecca Traub, Vito Colella

**Affiliations:** a Melbourne Veterinary School, Faculty of Science, University of Melbourne, Parkville, Victoria 3050, Australia; b National Centre for Animal Health, Serbithang, Thimphu 11001, Bhutan; c CityU Jockey Club College of Veterinary Medicine and Life Sciences, City University of Hong Kong, Kowloon, Hong Kong

**Keywords:** Zoonosis, Next-generation sequencing, MinION, *Ehrlichia*, *Dirofilaria* sp. hongkongensis, *Bartonella*, *Setaria tundra*, One Health

## Abstract

The diversity and prevalence of canine vector-borne pathogens (VBPs) in Bhutan have to date remained unexplored, whilst recent epidemiological surveys in other South Asian nations have found diseases caused by VBPs to be rife in local dog populations. Importantly, many of such VBPs can infect people as well, with a building body of evidence identifying potentially zoonotic rickettsial organisms infecting humans in Bhutan. Given the lack of data on canine pathogens in Bhutan we employed a suite of deep-sequencing metabarcoding methods using Oxford Nanopore Technologies' MinION™ device to holistically characterise the bacterial, apicomplexan and filarial worm blood-borne pathogens of dogs in the country's south. Of the 95 stray, owned and community dogs sampled 78% (95% CI = 69%–85%) were infected with at least one VBP. Pathogen species detected were highly diverse including the bacteria *Mycoplasma haemocanis* in 16% (95% CI: 10–24%), *Ehrlichia canis* in 4% (95% CI: 2–10%), *Anaplasma platys* in 2% (95% CI: 0.5–7%) of dogs as well as the zoonotic species *Bartonella clarridgeiae* in 1% (95% CI: 0.1–6%), a potentially novel *Bartonella* spp. and an *Ehrlichia chaffeensis*-like bacterium, both in 1% (95% CI: 0.1–6%) of dogs. The apicomplexan haemoparasites *Hepatozoon canis* in 62% (95% CI: 52–71%), *Babesia gibsoni* in 45% (95% CI: 36–55%) and *Babesia vogeli* in 3% (95% CI: 1–9%) of dogs were also detected. Finally, 5% (95% CI: 2–12%) of dogs were found to be infected with the filarioid *Acanthocheilonema reconditum* and 1% (95% CI: 0.1–6%) with zoonotic *Dirofilaria* sp. hongkongensis. One canine was found positive to the filarioid *Setaria tundra*, a species normally found infecting cervids. The elucidated diversity of VBP communities highlights the strength of assumption-free diagnostics, such as metabarcoding, in detecting rare, novel, and unexpected pathogens. This approach to identifying pathogen diversity is of critical importance when investigating regions and populations that have thus far been neglected, with the findings aiding the development of future One Health informed strategies for disease control.

## Highlights

• Metabarcoding assays are ideal for characterising pathogens from neglected regions
• Thirteen species of vector-borne pathogens (VBPs) were detected from Bhutanese dogs
• Zoonotic pathogens were detected including *Bartonella* spp. and *Dirofilaria* sp.
• Molecular surveillance is required to control VBPs in countries such as Bhutan

## 1 Introduction

Vector-borne pathogens (VBPs) i.e., those that are transmitted by arthropods, are a global and pervasive threat to human and animal health, capable of causing serious disease and death [[1], [2], [3]]. Many VBPs are also zoonotic, with domestic animals or wildlife acting as reservoirs that can facilitate the maintenance and transmission of such pathogens to humans [[4], [5], [6], [7]]. Dogs, for example, can be infected by bacterial VBPs, such as *Rickettsia felis* capable of causing flea-borne spotted fever in people, or filarial worms such as *Dirofilaria* sp. hongkongensis and *Brugia* spp. both also capable of producing zoonotic infections in humans [[8], [9], [10], [11], [12], [13]]. The close association pets, such as dogs, have with their owners may increase the chances of animal-to-human transmission whilst the animal's infection may go undetected as many zoonotic VBPs do not generate clinical signs in canines [[14], [15], [16]]. Moreover, many non-zoonotic VBPs can cause severe infections in canines, e.g., *Ehrlichia canis* the bacterial aetiological agent of canine monocytic ehrlichiosis, which can be lethal, or apicomplexan haemoparasites of the genus *Babesia* that can cause acute haemolytic anaemia [[17], [18], [19], [20], [21]].

The prevalence of canine VBPs is significantly increased in low and lower-middle income countries (LMICs) where free-roaming dogs are common and veterinary care may be limited or absent [4,14,22]. In LMICs like Bhutan, this situation is further compounded in many regions by a tropical climate that can be conducive to year-round ectoparasite survival, supporting sustained transmission and a burgeoning pathogen diversity [[22], [23], [24]]. In addition, according to the latest data available, the number of dogs in Bhutan is high relative to its human population of ~721,000 (2012), with an estimated 71,245 owned dogs and 48,379 stray dogs in the country as of 2012 [25]. Present-day numbers are likely to be significantly higher, given the predominance of Buddhism in Bhutan meaning culling of free-roaming dogs is prevented, although a nationwide capture-neuter-release program may have offset this to some degree [26].

Despite Bhutan's large population of dogs there is a paucity of data on canine VBPs within the country, whilst information on the epidemiology of zoonotic diseases in people is also limited. A serological survey from 2015 that tested a variety of domestic animals found that dogs had the highest levels of exposure to both spotted fever group (SFG) and typhus group (TG) *Rickettsia*, with 55% and 45% of 84 dogs seropositive, respectively [27]. Concurrent levels of exposure in humans at the same study site and sampling time were also high with a seroprevalence of 15.7% for SFG *Rickettsia* and 3.5% for TG *Rickettsia*, with the authors suggesting human rickettsial disease in the region may be linked and proportionate to that in animals [27,28]. Nonetheless, such data only represents the results of a couple of studies that have employed diagnostic methods that can only target exposure to a few VBP groups, with no species-level pathogen classification obtainable.

Given this dearth of relevant canine VBP data in Bhutan traditional molecular methods, such as conventional PCR (cPCR) and quantitative PCR (qPCR), that require a priori knowledge of a region's expected pathogens cannot be relied upon [29,30]. Instead, assumption-free diagnostic approaches must be used, such as next-generation sequencing (NGS) metabarcoding that can accurately amplify, sequence and classify all pathogens from a group of interest concurrently [31]. Such methods have recently shown great promise on Oxford Nanopore Technologies' (ONT) MinION™, a long-read sequencing device that can easily sequence full-length barcoding genes [32]. This technology has already been used to sequence the whole bacterial 16S ribosomal RNA (16S rRNA) gene, the apicomplexan 18S ribosomal RNA (18S rRNA) gene and the almost entire filarioid cytochrome oxidase I (COI) gene, providing species-level classification of all relevant haemopathogens, except for viruses [[32], [33], [34]]. Metabarcoding techniques excel at detecting rare, unexpected, or novel pathogens and have demonstrated a comparable sensitivity and specificity to traditional qPCR assays [32,33,35].

For zoonotic VBPs to be controlled basic information regarding pathogen diversity and prevalence in reservoir hosts must be elucidated, which can then inform future diagnostic strategies and aid in interventions that are informed by a One Health approach to disease control [22,31]. Taking this into consideration our study set out to utilise deep-sequencing metabarcoding for the holistic detection and characterisation of blood-borne bacterial, apicomplexan and filarial worm pathogens from stray, owned and community dogs in Southern Bhutan.

## 2 Materials and methods

### 2.1 Study sites and sample collection

**Fig. 1:**
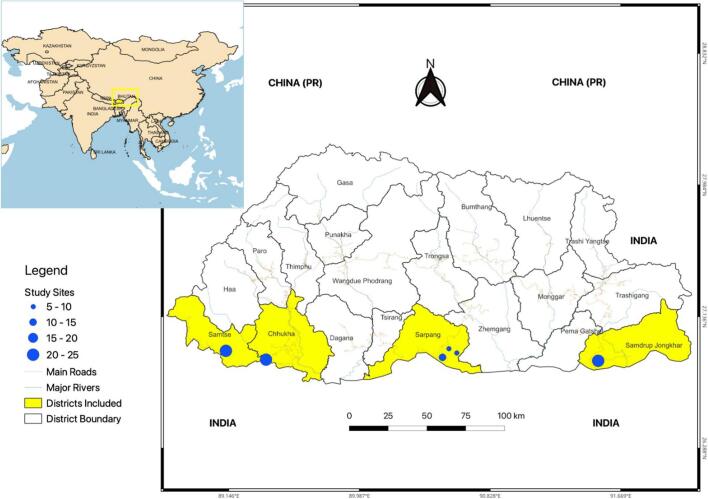
**Map of districts and field sites in Bhutan where canine samples were collected.** The size of the site circle is proportional to the number of samples taken. Map created in QGIS 3.4 via QGIS.org, 2021. QGIS Geographic Information System. QGIS Association.

For this proof-of-concept case study a total of 95 blood samples from stray, owned and community dogs were collected from study sites within four districts of Southern Bhutan; Chhukha (26.8592 N, 89.3848E) *n* = 25, SamdrupJongkhar (26.8030 N, 91.5025E) *n* = 24, Samtse (26.9038 N, 89.0991E) *n* = 21 and Sarpang (26.8651E, 90.2703 N) n = 25, see Fig. 1 for districts and exact locations sampled. Samples were collected in the months of April and May in 2022. For this initial pilot study sampling sites in the south of the country on the border with India (Fig. 1) were chosen due to the large free-roaming dog population, high density of human occupancy as well as warm and humid subtropical climate suitable for year-round maintenance of ectoparasites and VBP transmission [25,36]. Free-roaming dogs were randomly selected and sampled during the country's Nationwide Accelerated Dog Population Management and Rabies Control Program. Most of the free-roaming dogs sampled from all field areas lacked access to adequate veterinary care, including anti-parasiticidal treatments, desexing and vaccination. There were no exclusion criteria for dog sampling apart from whether the individual was too aggressive to capture and restrain.

Dogs were humanely restrained, and blood was only taken following informed consent from the animal's owner in the case of community or pet dogs. A two-millilitre sample of whole blood was collected by a veterinarian through cephalic venipuncture into an anti-coagulation EDTA tube and temporarily kept on ice. Blood samples were stored frozen at -20 °C upon return to the nearest laboratory.

Relevant dog metadata including age, sex, breed, whether neutered, reproductive status and sampling location coordinates, were recorded and findings of clinical examinations performed at time of sample collection were noted. Clinical examinations were conducted by a qualified veterinarian with body score taken [37], dog demeanour, haircoat and mucous membrane condition and respiration rate all visually evaluated. The presence of ectoparasites, such as fleas, ticks and lice, was also recorded.

Field work in Bhutan was conducted under the Livestock Technical Advisory Committee, Department of Livestock, Bhutan ethics permit: DOL/GEN-23/2020-2021/2202 granted 21.05.2021.

### 2.2 DNA extraction

Whole blood samples were couriered frozen to the University of Melbourne Veterinary School, Australia for processing. A total of 200 μl of defrosted whole blood was extracted using the DNeasy Blood & Tissue Kit (Qiagen, Hilden, Germany) using the manufacturer's protocol with a 30-min proteinase K digestion at 56 °C and two final elution steps, the first in 30 μl and second in 20 μl (50 μl total eluent). Extracted DNA was kept at -20 °C until it was required. All DNA extracts were quantified using a Qubit™ 4 Fluorometer (Thermo Fisher Scientific, Massachusetts, USA) using the dsDNA HS assay kit.

### 2.3 Pre-screening conventional PCR to select samples for apicomplexan and filarial worm metabarcoding

To prevent deep-sequencing of apicomplexan and filarial worm negative samples pre-screening cPCR assays were used to assess whether a given sample was positive to a relevant pathogen from these groups. If a sample showed a band on a gel, then it was taken forward for NGS metabarcoding. Given the ubiquity of bacteria even in healthy canine blood [[38], [39], [40]], this initial cPCR pre-screening method could not be employed for the bacterial metabarcoding work.

For apicomplexan metabarcoding pre-screening 20 μl PCRs were conducted using OneTaq® 2× Master Mix with Standard Buffer (New England Biolabs, Ipswich, USA), 1 μl each of primers BT1-F (5’- GGTTGATCCTGCCAGTAGT -3’) and 18SApiR_Mod (5’- GGATCACTCRATCGGTAGGAG -3’) both at 10 μM as well as 2 μl of template DNA [33,41]. Apicomplexan pre-screening PCRs were conducted on a T100™ Thermal Cycler (Bio-Rad, Hercules, USA) using the following conditions: 1 cycle of 95 °C for 40 s, 40 cycles of 95 °C for 30 s, 58 °C for 45 s and 68 °C for 1 min 20 s, with a final extension of 68 °C for 5 min. Expected product size was ~1,600 bp.

Pre-screening for filarial worm metabarcoding was carried out in 25 μl PCRs using 1 μl each of the COIfilF and COIfilR primers from [42] and 2 μl of template DNA. Thermocycling conditions were modified to: 1 cycle of 95 °C for 40 s, 40 cycles of 95 °C for 40 s, 50 °C for 45 s and 68 °C for 45 s, with a final extension of 68 °C for 5 min. Expected product size was ~650 bp.

Positive and negative controls were run for all pre-screening cPCRs. Gel electrophoresis using 1.5% agarose gels was conducted on all PCR products at 90 V for 45 min, with bands visualised on a ChemiDoc™ Imaging System (Bio-Rad, Hercules, USA). If a band was visualised then the samples were sent forward for metabarcoding, however amplicons generated by this pre-screening cPCR were not used for metabarcoding library preparation, instead positive samples were processed from the beginning as per the protocol in the proceeding sections.

### 2.4 Metabarcoding of samples to elucidate VBP communities

The PCR Barcoding Expansion 1-96 (EXP-PBC096) with ONT’ Ligation Sequencing Kits (SQK-LSK110) were used to conduct the library preparation for deep-sequencing on the MinION Mk1B device (Oxford Nanopore Technologies, Oxford, UK). Metabarcoding of the bacterial 16S rRNA gene was conducted for all samples (*n* = 95), whilst metabarcoding for the apicomplexan 18S rRNA and filarial worm COI gene was only conducted on those samples found positive by cPCR, *n* = 70 and *n* = 6, respectively. Library preparation was carried out exactly as per the relevant publication for apicomplexan [33] and filarial worm [34] metabarcoding. For bacterial metabarcoding the previously developed method [32] was adapted to permit multiplexing of up to 96 samples by using the ‘Ligation sequencing amplicons - PCR barcoding (SQK-LSK110 with EXP-PBC096)’ version: PBAC96_9114_v110_revK_10Nov2020 protocol from ONT. In brief, first step PCRs were conducted in 25 μl reactions using 3 μl of blood extracted template DNA and 1 μl each of the primers Bact-16S-ONT-7F: 5’- TTTCTGTTGGTGCTGATATTGCAGAGTTTGATCMTGGCTCAG -3’.

Bact-16S-ONT-1492R: 5’- ACTTGCCTGTCGCTCTATCTTCCGGTTACCTTGTTACGACTT -3’ (underlined sequences are the relevant ONT adapters). PCRs used the following conditions: 1 cycle of 95 °C for 1 min, 22 cycles of 95 °C for 30 s, 55 °C for 45 s and 65 °C for 2 min, with a final extension of 65 °C for 5 min. PCR product was cleaned using a 1× ratio of NucleoMag NGS Clean-up and Size Select Beads (Macherey-Nagel, Duren, Germany). Second step PCRs were conducted in 50 μl reactions as per ONT’ protocol followed by a clean-up step using a 0.65× ratio of beads. Next, 2 μl of each cleaned PCR product were pooled together and concentrated down using a 2× ratio of NucleoMag beads, washed twice with 75% ethanol, eluted in 50 μl of water, quantified and then diluted to the necessary concentration. Final library preparation steps (DNA repair and end-prep, adapter ligation, etc.) were carried out as per the ONT’ protocol. Two batches of pooled sample amplicons were processed, a 96-sample batch (56 fmol loaded onto a new R9.4.1 flow cell) and an 8-sample batch (20 fmol loaded onto a used R9.4.1 flow cell). For the apicomplexan metabarcoding 54 fmol of pooled library was added to a new flow cell, whilst for the filarial worm metabarcoding 29.3 fmol of library was loaded onto a previously used and flow cell. Any re-used flow cells underwent a DNAse clean-up using the EXP-WSH004 Flow Cell Wash Kit (Oxford Nanopore Technologies, Oxford, UK).

All metabarcoding batches were run with positive and negative controls. For the bacterial metabarcoding nine controls were used across both batches, three no template PCR negative controls, (water), three DNA extraction negative controls (reagent-only DNA extracts) and three positive controls that were comprised of a uniquely identifiable gBlock synthetic DNA strand, see [32]. For apicomplexan metabarcoding two PCR negative controls, one DNA extraction control and two positive controls [33] were used, whilst for the filarial worm metabarcoding one of each control type was used [34].

Nanopore sequencing was conducted on a MinION Mk1B device using a Legion 7i Gen 6 laptop (Lenovo, Quarry Bay, Hong Kong) for between 19 and 40 h, depending on the sequencing batch size [[32], [33], [34]]. FAST5 files were then base-called using the super high accuracy base-calling model with barcode removal using Guppy version 6.4.6.

All nanopore sequencing data produced in this study are available from the NCBI BioProject database BioProjectID: PRJNA1066489; specifically, Sequence Read Archive (SRA) accessions SRR27674540 to SRR27674643 for bacterial 16S rRNA gene data, accessions SRR27674753 to SRR27674827 for apicomplexan 18S rRNA gene data and accessions SRR27674644 to SRR27674652 for filarial worm COI gene data.

### 2.5 Bioinformatics

All bioinformatic processing was conducted using the NanoCLUST pipeline as it corrects for the error rate of R9.4.1 flow cells by construction of accurate barcoding gene consensus sequences [43]. For bacterial metabarcoding data processing all consensus sequences were generated as per previously identified parameters [32] and classified against the Ribosomal Database Project (RDP) Release 11 database. For apicomplexan and filarial worm metabarcoding the NanoCLUST parameters chosen, and in-house reference taxonomic databases utilised, were as per [33] and [34], respectively.

Read thresholds to determine whether a sample is positive for a given VBP were calculated as per [35] using a uniquely identifiable positive control construct of the 16S rRNA gene from the bacterium *Aliivibrio fischeri*. Positive control reads found in samples other than the positive control and reads from known VBP found in negative controls informed the read cut-off threshold for a given sequencing batch. Read thresholds for a given batch were the highest read-count of a positive control sequence in a non-positive control sample or confirmed VBP read-count in a negative control sample; whichever was larger.

### 2.6 Statistical analyses

The 95% confidence intervals (CIs) for the frequency of detection of given pathogens were calculated using the Wilson score interval via the open-source software Epitools (https://epitools.ausvet.com.au).

Chi-square (χ^2^) statistical methods were calculated using IBM SPSS Statistics 28.0.1.0 (SPSS, Chicago, USA) and used to compare measured categorical variables against NGS positivity for VBPs. Variables included in the analyses were study site, dog age (juvenile ≤12 months or adult ≥13 months), sex, husbandry (stray, owned or community), presence of ectoparasites, haircoat condition, body score, dog demeanour, mucous membrane condition and respiration rate for example panting. Variables with a significance of *P* < 0.2 found via univariate analysis were then taken forward and included in a multiple logistic regression model.

Next, the multiple logistic regression model was built in GraphPad Prism 10.0.0 (GraphPad Software, San Diego, USA) and was used to identify variables associated with VBP positivity. After the first iteration of this model the least significant variable was removed using iterative backward elimination of variables until the final multivariable model was obtained. Associations were considered statistically significant if *P* < 0.05 or close to it. The best-fit model was selected using Akaike's corrected Information Criterion value, multicollinearity was checked using variance inflation factors to check for strongly dependent predictors. Area under the receiver operating characteristic (ROC) curve was used to measure the ability of the model in classifying negative and positive events.

### 2.7 Phylogenetic analyses

For three of the unusual taxa identified through our metabarcoding protocols, i.e., *Dirofilaria* sp. hongkongensis, *Ehrlichia chaffeensis*-like bacterium and *Setaria tundra*, phylogenetic trees were constructed. Relevant bacterial 16S rRNA and filarial worm mitochondrial COI genes were downloaded from GenBank, imported into Geneious Prime® 2023.2.1 (Biomatters, Auckland, New Zealand), and aligned with the relevant sequences obtained from our metabarcoding approach, using MAFFT. Alignments were exported as FASTA files which were subsequently converted to the NEXUS format with MEGA 11 (version 11.0.13) [44]. The best nucleotide substitution model for each alignment was determined with maximum likelihood analysis, including 1st, 2nd, and 3rd codon positions in MEGA 11. The model with the lowest Bayesian Information Criterion (BIC) value was selected for phylogenetic inference.

For Bayesian phylogenetic inference (BI) selected FASTA alignments were converted to the NEXUS format for MrBayes [45], that includes a code block with instructions for Bayesian inference with Mesquite: a modular system for evolutionary analysis (version 3.70) software [46]. Bayesian phylogenetic inference was performed using MrBayes 3.2.7 [47] separately on each alignment. Each BI was performed with two million Markov Chain Monte Carlo (MCMC) generations, sampling every 100th generation with four chains by allowing for transitions and transversions with gamma-distributed rates. Phylogenetic inference by the Neighbour-Joining (NJ) distance method was performed in MEGA 11 (version 11.0.13) for all three alignments. The NJ analysis was performed with 2000 bootstrap replications using the relevant model, based on best nucleotide substitution models as determined above, including both transition and transversions with gamma-distributed rates. Trees outputted by MrBayes were imported into FigTree (version 1.4.4.) and then Adobe Illustrator version 27.3.1 (Adobe, San Jose, USA) for editing to improve clarity.

## 3 Results

### 3.1 Sample collection

A total of 95 dogs were sampled from four field sites. The demographic composition of all canines sampled, except for those located in Samtse, was 39 (41%) females, 35 males (37%) and 21 (22%) unreported, with most dogs (80% of those with metadata) between one and seven years of age (Table 1). All dogs were mongrels with no pure breeds encountered. Most dogs had a normal skin condition, body score, demeanour, mucous membrane condition and respiration, however ectoparasite infestation was common with 31 dogs (42% of those with metadata) found with ticks or fleas (Table 1).

**Table 1:** **Canine demographic data, health condition and presence of ectoparasites for dogs sampled in Samdrup Jongkhar, Sarpang, and Chukha.** Demographic data of dogs from Samtse was not obtained. Under skin condition * refers to skin being rough, with erythematic, alopecia or pruritus. Under respiratory condition ^ refers to panting or shallow breathing. For dogs with ectoparasites † refers to F for fleas and T for ticks.

**Study Area**	**Samdrup Jongkhar** **(*n* = 24)**	**Sarpang** **(*n* = 25)**	**Chukha (n = 25)**	**Total (*n* = 74)**
**Sex**	**n (%)**	**n (%)**	**n (%)**	**n (%)**
Female	15 (63)	11 (44)	13 (52)	39 (53)
Male	9 (38)	14 (56)	12 (48)	35 (47)
**Age (months)**				
≤ 6	0 (0)	0 (0)	3 (12)	3 (4.)
≥ 7 to ≤12	1 (4)	2 (8)	4 (16)	7 (9)
≥ 13 to ≤84	20 (83)	23 (97)	16 (84)	59 (80)
≥ 85	3 (13)	0 (0)	2 (8)	5 (7)
**Ownership**				
Owned	5 (21)	18 (72)	0 (100)	23 (31)
Community	0 (0)	0 (100)	4 (16)	4 (5)
Stray	19 (79)	7 (28)	21 (84)	47 (64)
**Skin Condition**				
Normal	24 (100)	25(100)	8 (32)	57 (77.03)
Abnormal*	0 (0)	0 (0)	17 (68)	17 (23)
**Body Score**				
< 20% Ideal Weight	0 (0)	5 (20)	2 (8)	7 (9)
Ideal Weight	24 (100)	20 (80)	23 (92)	67 (91)
**Demeanour**				
Alert	24 (100)	25 (100)	21 (84)	70 (95)
Depressed	0 (0)	0 (0)	4 (16)	4 (5)
**Mucous Membranes**				
Normal	24 (100)	25 (100)	21 (84)	70 (95)
Pale	0 (0)	0 (0)	4 (16)	4 (5)
**Respiratory Condition**				
Normal	24 (100)	25 (100)	22 (88)	71 (96)
Abnormal^	0 (0)	0 (0)	3 (12)	3 (4)
**Ectoparasites**				
Present	15 (63)	1 (4)	15 (60)	31 (42)
Ectoparasite Type†	F: 15 (100)	T: 1 (100)	F: 10 (67), T: 5 (33)	F: 25 (81), T: 6 (19)
Absent	9 (38)	24 (96)	10 (40)	43 (58)

### 3.2 Bioinformatic processing

Four separate sequencing runs were used to sequence the bacterial, apicomplexan, and filarial worm pathogens from 96 Bhutanese canine blood samples (Table 2). Sequencing was stopped after flow cell death to ensure the maximum quantity of data was accrued. Previously used flow cells were only utilised for smaller sequencing batches, such as the second batch of bacterial samples, or the filarial worms. A flow cell was only re-used for a sequencing batch after appropriate flow-cell washing and if it had never previously been used for metabarcoding of the relevant pathogen group. For example, the flow cell used for filarial worm sequencing had never previously had filarial worm amplicons run on it, to eliminate the possibility of DNA carry-over from a prior sequencing run. The base-called pass:fail ratio was better for flow cells that had not been used previously, with a Q-score of ≥8 categorised as a pass. The total raw and processed reads were also better for new flow cells (Table 2), however mean sequencing depth was determined as sufficient for all sequencing batches when compared to reference depths determined by the relevant assay's original publication [[32], [33], [34]].

**Table 2:** Nanopore sequencing batch performance pre- and post-read filtering. All 96 canine samples were sequenced using the bacterial assay, whilst a pre-screening conventional PCR assay was used to only deep-sequence samples that were positive for apicomplexan or filarial worm pathogens. Flow cell state refers to whether a new flow cell was used or if an old flow cell that had been washed using a DNAse treatment was employed. Base-called nucleotides were only given a pass status if they met a Q-score of ≥8. Processed reads refer to those that were outputted by the NanoCLUST bioinformatic pipeline, i.e., the final metabarcoding dataset.

**Parameter**	**Bacteria**		**Apicomplexans**	**Filarial Worms**
	**Batch 1**	**Batch 2**	**Only Batch**	**Only Batch**
**Total samples multiplexed**	96	8	75	9
**Controls (positive, negative)**	6 (2,4)	3 (1,2)	5 (2,3)	3 (1,2)
**Flow cell state**	New	Used - washed	New	Used - washed
**Duration (hrs)**	24	38	40	19
**Total data (GB)**	87.5	34.3	83.2	5.24
**Base-called pass:fail ratio (GB)**	5.45:1.37	1.58:0.98	4.45:1.85	0.19:0.01
**Total raw reads**	2,926,655	471,773	2,570,922	172,206
**Mean raw reads (S.E.)**	30,486 (6630)	58,972 (13,132)	34,279 (4174)	19,134 (7936)
**Total processed reads**	2,695,079	400,816	2,194,581	131,559
**Mean processed reads (S.E.)**	28,369 (6334)	50,102 (12,308)	31,351 (3362)	14,618 (6307)

### 3.3 VBP Metabarcoding

**Fig. 2:**
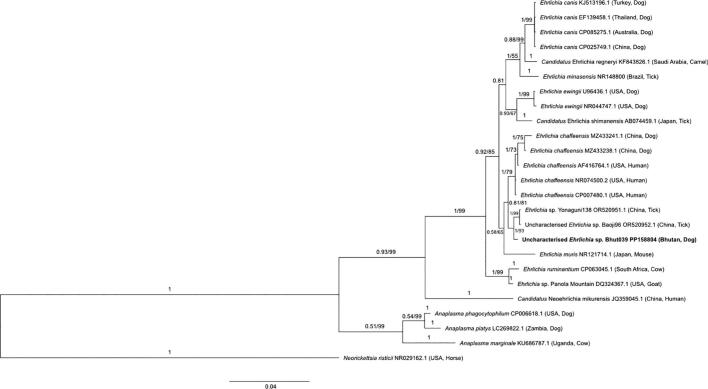
**Phylogenetic relationship of the uncharacterised *Ehrlichia* taxon (bold) from the blood of one Bhutanese dog alongside representative sequences from across the family Ehrlichiaceae.** Phylogenetic inference was made using a Bayesian and neighbour-joining distance method for a 1357 bp segment of the 16S rRNA gene. Posterior probability values and bootstrap support (where available) for tree branches are indicated, with *Neorickettsia risticii* used as an outgroup. The taxonomic label in bold indicates the *Ehrlichia* sequence (accession # PP158804) generated in this study.

Read counts for bacteria, apicomplexans and filarial worms that are known or putative pathogens were extracted from the overarching datasets and compiled. These were the bacteria *Anaplasma platys, Bartonella clarridgeae, Ehrlichia canis,* an *Ehrlichia chaffeensis*-like bacterium (Fig. 2)*, Mycoplasma haemocanis* and *Staphylococcus pseudintermedius*, the apicomplexans *Babesia gibsoni, Babesia vogeli* and *Hepatozoon canis* as well as the filarial worms *Acanthocheilonema reconditum*, *Dirofilaria* sp. hongkongensis and *Setaria tundra*, see Table 3 for the frequency of detection of these pathogens by field site. In addition, one sample had bacterial sequences that could only be confidently classified down to a genus level for *Bartonella* as they obtained a top hit in NCBI's GenBank to both *Bartonella chomelii* (identity 99.79% to accession number JN646644.1) and *Bartonella schoenbuchensis* (identity 99.72% to accession number NR_025410.1). Therefore, this taxon is hereon referred to as *Bartonella* spp. and has been uploaded to NCBI's GenBank under accession number PP158805 (also in Table 3).

**Table 3:** Number of pathogen positive dogs (+) and frequency of detection (%) with 95% confidence intervals (CI) that were found for both total infections (single + coinfected) and coinfected only. The frequency of detection is shown by field site and across all field sites. Table shows data from blood detected organisms that are confirmed pathogens of canines or other animals.

**Study Sites**	**Chhukha (n** **= 25)**		**Samdrup Jongkhar (*n*** **= 24)**		**Samtse (*n*** **= 21)**		**Sarpang (*n*** **= 25)**		**All Sites (n** **= 95)**	
	**+**	**% (CI)**	**+**	**% (CI)**	**+**	**% (CI)**	**+**	**% (CI)**	**+**	**% (CI)**
Single & coinfections										
*Acanthocheilonema reconditum* (AR)	4	16 (6–35)	0	0 (0–14)	0	0 (0–15)	1	4 (1−20)	5	5 (2−12)
*Anaplasma platys* (AP)	1	4 (1–20)	0	0 (0–14)	0	0 (0–15)	1	4 (1–20)	2	2 (0.5 - 7)
*Babesia gibsoni* (BG)	14	56 (37–73)	2	8 (2–26)	8	38 (21–59)	19	76 (57–89)	43	45 (36 - 55)
*Babesia vogeli* (BV)	1	4 (1–20)	2	8 (2–26)	0	0 (0–15)	0	0 (0−13)	3	3 (1 - 9)
*Bartonella clarridgeae* (BC)	0	0 (0–13)	1	4 (1–20)	0	0 (0–15)	0	0 (0–13)	1	1 (0.1 - 6)
*Bartonella* spp. (BS)	1	4 (1–20)	0	0 (0–14)	0	0 (0–15)	0	0 (0–13)	1	1 (0.1 - 6)
*Dirofilaria* sp. hongkongensis (DH)	0	0 (0–13)	1	4 (1–20)	0	0 (0–15)	0	0 (0–13)	1	1 (0.1 - 6)
*Ehrlichia canis* (ECa)	2	8 (2–25)	0	0 (0–14)	0	0 (0–15)	2	8 (2–25)	4	4 (2−10)
*Ehrlichia chaffeensis-*like bacterium (ECh)	0	0 (0–13)	0	0 (0–14)	0	0 (0–15)	1	4 (1–20)	1	1 (0.1 - 6)
*Hepatozoon canis* (HC)	10	40 (23–59)	14	58 (39–76)	12	57 (37–76)	23	92 (75–98)	59	62 (52 - 71)
*Mycoplasma haemocanis* (MH)	7	28 (14–48)	4	17 (7–36)	3	14 (5–35)	1	4 (1–20)	15	16 (10 - 24)
*Setaria tundra* (ST)	0	0 (0–13)	0	0 (0–14)	0	0 (0–15)	1	4 (1–20)	1	1 (0.1 - 6)
*Staphylococcus pseudintermedius* (Sp)	1	4 (1–20)	0	0 (0–14)	0	0 (0–15)	0	0 (0–13)	1	1 (0.1 - 6)

**Coinfections**										
AP & AR	1	4 (1–20)	0	0 (0–14)	0	0 (0–15)	0	0 (0–13)	1	1 (0.1 - 6)
AR & BG	1	4 (1–20)	0	0 (0–14)	0	0 (0–15)	0	0 (0–13)	1	1 (0.1 - 6)
BG & BV	1	4 (1–20)	0	0 (0–14)	0	0 (0–15)	0	0 (0–13)	1	1 (0.1 - 6)
BG & HC	4	16 (6–35)	1	4 (1–20)	6	29 (14–50)	15	60 (41–77)	26	27 (19 - 37)
BG & MH	1	4 (1–20)	0	0 (0–14)	0	0 (0–15)	0	0 (0–13)	1	1 (0.1 - 6)
BV & HC	0	0 (0–13)	2	8 (2–26)	0	0 (0–15)	0	0 (0–13)	2	2 (0.5 - 7)
ECh & HC	0	0 (0–13)	0	0 (0–14)	0	0 (0–15)	1	4 (1–20)	1	1 (0.1 - 6)
HC & Mh	0	0 (0–13)	1	4 (1–20)	1	5 (1−23)	0	0 (0–13)	2	2 (0.5 - 7)
MH & SP	1	4 (1–20)	0	0 (0–14)	0	0 (0–15)	0	0 (0–13)	1	1 (0.1 - 6)
AR & BG & ECa	1	4 (1–20)	0	0 (0–14)	0	0 (0–15)	0	0 (0–13)	1	1 (0.1 - 6)
BG & ECa & HC	0	0 (0–13)	0	0 (0–14)	0	0 (0–15)	1	4 (1–20)	1	1 (0.1 - 6)
BG & ECa & MH	1	4 (1–20)	0	0 (0–14)	0	0 (0–15)	0	0 (0–13)	1	1 (0.1 - 6)
BG & HC & MH	3	12 (4–30)	1	4 (1–20)	1	5 (1–23)	1	4 (1–20)	6	6 (3−13)
AP & BG & ECa & HC	0	0 (0–13)	0	0 (0–14)	0	0 (0–15)	1	4 (1–20)	1	1 (0.1 - 6)
AR & BG & BS & HC	1	4 (1–20)	0	0 (0–14)	0	0 (0–15)	0	0 (0–13)	1	1 (0.1 - 6)
AR & BG & HC & ST	0	0 (0–13)	0	0 (0–14)	0	0 (0–15)	1	4 (1–20)	1	1 (0.1 - 6)
**Total coinfections**	15	60 (41 - 77)	5	21 (9 - 40)	8	38 (21 - 59)	20	80 (61 - 91)	48	51 (41 - 60)
**Total pathogen positive**	19	76 (57 - 89)	18	75 (55 - 88)	14	67 (45 - 83)	23	92 (75 - 98)	74	78 (69 - 85)
**Total pathogen negative**	6	24 (11 - 43)	6	25 (45 - 12)	7	33 (55 - 17)	2	8 (25 - 2)	21	22 (15 - 31)

Across all study sites 78% of dogs (95% CI = 69% - 85%) were found to be infected with at least one of these thirteen pathogens, whilst 51% (95% CI = 41% - 60%) of all dogs had a coinfection comprising two or more VBPs (Table 3). The frequency of detection of a dog having at least one pathogen was high across all field sites, with Sarpang having the most infected canines at 92% (95% CI = 75% - 98%), followed by Chhukha at 76% (95% CI = 57% - 89%), Samdrup Jongkhar at 75% (95% CI = 55% - 88%) and Samtse having the lowest number of pathogen positive canines at 67% (95% CI = 45% - 83%) see Table 3.

From the confirmed pathogens detected by our metabarcoding assays apicomplexans such as *H. canis* (62%; 95% CI = 52% - 71%) and *B. gibsoni* (45%; 95% CI = 36% - 55%) were the most common detected, whilst *M. haemocanis* (16%; 95% CI = 10% - 24%) was the most common bacterial pathogen detected, followed by *E. canis* (4%; 95% CI = 2% - 10%). From the filarial worms detected *A. reconditum* was the most common, identified in 5% (95% CI = 2% - 12%) of samples (Table 3). The most frequently detected VBP coinfection comprised of a *B. gibsoni* and *H. canis* coinfection (27%; 95% CI = 19% - 37%), followed by *B. gibsoni*, *H. canis* and *M. haemocanis* triple infections (6%; 95% CI = 3% - 13%) see Table 3.

### 3.4 Statistical and risk indicator analyses

Univariate analysis identified dog age, i.e. being an adult, dog sex, i.e. being a male, and the presence of ectoparasites as having a statistically significant association with NGS positivity for a VBP (*P* < 0.05). Study site location and mucous membrane condition were also found associated with NGS positivity for a VBP at the *P* < 0.2 level. These variables were all included in the multiple logistic regression model.

Our multiple logistic regression model identified three variables as being predictors of VBP positivity in Bhutanese dogs (Table 4). Dog age, i.e. being a juvenile was associated with a decreased odds of being VBP positive when other variables were kept constant. Dog sex, i.e. being male was associated with an increased odds of being VBP positive at a *P* value approaching 0.05 (*P* = 0.069) when all other variables were kept constant, whilst the presence or absence of ectoparasites was also found to be a significant predictor of VBP positivity (Table 4).

**Table 4:** Parameter estimates and odds ratios (95% profile likelihood) for vector-borne pathogen positivity in 74 dogs from Bhutan.

**Parameter estimates**	**Estimate**	**Standard error**	**Odds ratios**	***P* value**
Intercept	2.316	0.706		
Age [Juvenile]	-2.425	0.894	0.09 (1.01-0.47)	0.007
Sex [Male]	1.5	0.825	4.48 (1.01-28.46)	0.069
Presence of ectoparasites	-1.632	0.7617	0.20 (0.04-0.79)	0.032

### 3.5 Phylogenetic analyses

A 1357 bp stretch of the 16S rRNA gene was used to place the *Ehrlichia chaffeensis*-like sequence from one Bhutanese dog within a phylogenetic tree (Fig. 2). Our Bhutanese sequence (accession # PP158804) was most similar to two sequences in GenBank from *Ehrlichia* species identified in ticks collected from grassland environments within China (100% query cover and 99.65% identity to OR520951.1 and OR520952.1). Nonetheless, subsequent top hits were to *E. chaffeensis* (100% query cover and 99.3% identity to NR074500.2, amongst others). These results were corroborated through 16S rRNA gene-based identification using the curated EzBioCloud Database (https://www.ezbiocloud.net). Through phylogenetic analysis we observed that our *Ehrlichia* sequence from a Bhutanese dog clusters within a well-supported clade with both the uncharacterised *Ehrlichia* from Chinese ticks and the characterised *E. chaffeensis* from dogs and humans in China and the USA (Fig. 2).

**Fig. 3:**
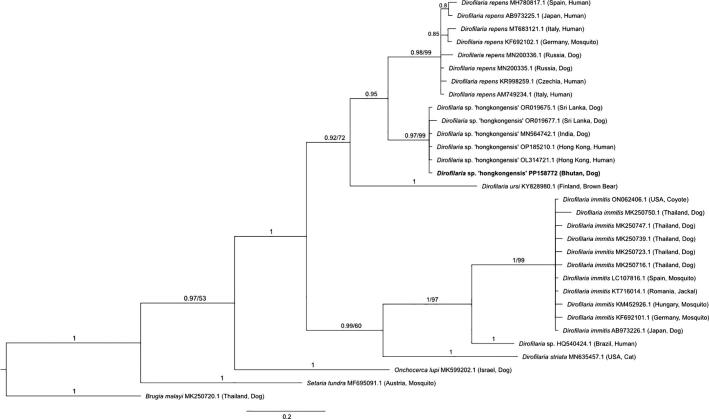
**Phylogenetic relationship of *Dirofilaria*** sp. **hongkongensis (bold) from the blood of a Bhutanese dog alongside representative sequences from across the families Onchocercidae and Setariidae.** Phylogenetic inference was made using a Bayesian and neighbour-joining distance method for a 465 bp segment of the filarial worm COI gene. Posterior probability values and bootstrap support (where available) for tree branches are indicated, with *Brugia malayi* used as an outgroup. The taxonomic label in bold indicates the *Dirofilaria* sp. hongkongensis sequence (accession # PP158772) generated in this study.

A 465 bp stretch of the filarial worm COI gene was used to build a phylogenetic tree and confirm the identity of one sequence from a Bhutanese dog as belonging to *Dirofilaria* sp. hongkongensis (Fig. 3). The sequence from our study (accession # PP158772) was most similar to a sequence from *Dirofilaria* sp. hongkongensis extracted from a human in India (100% query cover and 99.85% identity to NC_031365.1, amongst others). Our *Dirofilaria* sp. hongkongensis sequence from Bhutan clusters with good support (posterior probability of 0.97 and bootstrap support of 99%) to a variety of other *Dirofilaria* sp. hongkongensis sequences from Hong Kong, India, and Sri Lanka, whilst also clustering separately to the closely related *D. repens* (Fig. 3).

**Fig. 4:**
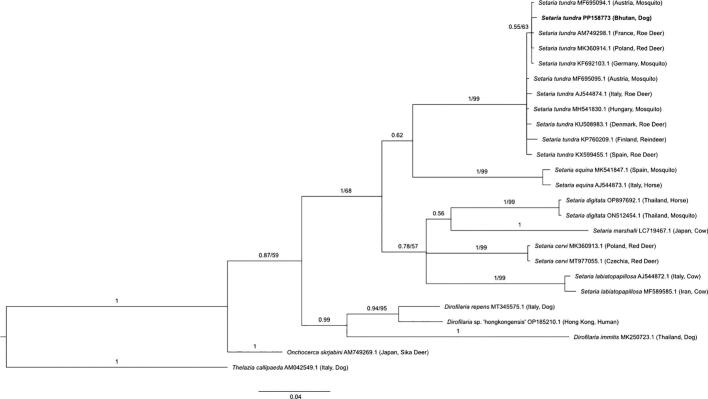
**Phylogenetic relationship of *Setaria tundra* (bold) from the blood of one Bhutanese dog alongside representative sequences from across the families Onchocercidae, Setariidae and Thelaziidae.** Phylogenetic inference was made using a Bayesian and neighbour-joining distance method for a 577 bp segment of the filarial worm COI gene. Posterior probability values and bootstrap support (where available) for tree branches are indicated, with *Thelazia callipaeda* used as an outgroup. The taxonomic label in bold indicates the *Setaria tundra* sequence (accession # PP158773) generated in this study.

A 577 bp stretch of the filarial worm COI gene was used to build a phylogenetic tree and confirm the identity of one sequence from a Bhutanese dog as belonging to *S. tundra* (Fig. 4). The sequence from our study (accession # PP158773) was most similar to a *S. tundra* extracted from a red deer in Poland (100% query cover and 99.85% identity to MK360914.1, amongst others). The *S. tundra* sequence we obtained from a Bhutanese dog clustered with good support (posterior probability of 1.00 and bootstrap support of 99%) to a variety of *S. tundra* sequences from deer and mosquitoes from numerous European countries, whilst also clustering separately to other *Setaria* species, such as *Setaria equina*, amongst others (Fig. 4).

### 3.6 Detection of putative pathogens

A high diversity of bacterial 16S rRNA gene sequences were detected from the canine samples collected from across Bhutan. Many of these detected bacteria were likely environmental or from the skin of the dog that may have contaminated the blood sample at the point of sampling. However, some bacterial species that are implicated as aetiological agents of disease in both canines and humans were also observed, including many that are not vector-borne. These putative pathogens were extracted from the overall data set and further characterised using NCBI's BLASTn to attain accession numbers and additional information (Table 5).

**Table 5:** BLASTn results for putative bacterial pathogens detected by the bacterial metabarcoding assay across all 95 Bhutanese dogs. The 'Dogs infected' column includes the frequency of detection (%) with 95% confidence intervals (CI) within parentheses.

**Putative bacterial pathogen**	**NCBI accession no.**	**Query cover**	**Identity**	**Length (bp)**	**No. of reads**	**Dogs infected** **(%: 95 CI)**
*Achromobacter xylosoxidans*	CP043820.1	100	99.8	1,485	316 & 75,219	2 (2%: 0.5 - 7)
*Brucella pseudogrignonensis*	CP015776.1	100	99.9	1,432	631 & 32	2 (2%: 0.5 - 7)
*Helicobacter canis*	KJ534297.1	99	98	1,608	580	1 (1%: 0.2 - 6)
*Kurthia gibsoni*	EF032677.1	100	99.7	1,505	592 & 63	2 (2%: 0.5 - 7)

## 4 Discussion

Here we demonstrate that the use of metabarcoding assays can uncover a hyper-diverse range of pathogenic organisms including potentially novel species from geographical areas and populations of animals that have thus far been neglected. To the best of the authors' knowledge no such epidemiological studies that employ multipronged molecular techniques have been used to characterise the pathogens of dogs in Bhutan before, making the identification of all thirteen confirmed pathogens newly identified in the country. Moreover, no research has demonstrated such high levels of blood-borne pathogen frequency of occurrence in Bhutanese dogs before, with as many as 78% of dogs infected with at least one confirmed pathogen and at least 42% of dogs infested with ectoparasites, such as ticks and fleas. Importantly, many of the VBPs detected in canines from Bhutan are known to be zoonotic and capable of generating potentially severe disease in people, including *Bartonella clarridgeae*, *Dirofilaria* sp. hongkongensis and an *Ehrlichia chaffeensis*-like bacterium [[48], [49], [50], [51]], with infected dogs in the country potentially representing a previously unidentified reservoir of these pathogens.

From our phylogenetic analyses (Fig. 2) we confirmed the identification of *E. chaffeensis* or an *Ehrlichia chaffeensis*-like bacterium from one dog. This analysis identified our sequence as clustering most closely to an *Ehrlichia* species identified in ticks from China, however these sequences could represent intraspecific variation within *E. chaffeensis* at the 16S rRNA gene given their close clustering with confirmed *E. chaffeensis* sequences (Fig. 2). Moreover, the nucleotide sequence similarity across the near complete 16S rRNA gene between the sequence we found from one Bhutanese dog and *E. chaffeensis* reference sequences in GenBank is 99.37%, higher than the 98.65% sequence similarity defined as a threshold to differentiate two bacterial species [52]. The possible identification of *E. chaffeensis* is important given that this pathogen is zoonotic and can cause a long-term infection in canines without any observable clinical signs of disease, although in some dogs pathogenesis is observed, including petechiae and thrombocytopaenia [53,54]. In contrast, *E. chaffeensis* infection in people causes human monocytic ehrlichiosis (HME) which can quickly become life-threatening, with infected individuals presenting with a spectrum of clinical signs from fever through to multiorgan failure and potentially death [51,55,56]. Whilst data on the prevalence of *E. chaffeensis* infections from dogs or humans in Asia are relatively rare, there are reports of this pathogen from human clinical samples from China and from canines in China, South Korea, and potentially from Japan as well, where suspected vectors are tick species such as *Haemaphysalis longicornis* [54,55,[57], [58], [59]].

Zoonotic *B. clarridgeae* and another *Bartonella* taxon that could not be identified to a species level were also found infecting one dog each, with the former bacterial pathogen known to be one of the aetiological agents of cat scratch disease (CSD), in conjunction with the more commonly encountered *Bartonella henselae* [60,61]. *Bartonella clarridgeae* has been identified infecting canines within Asia before and although this bacterial species typically does not cause serious disease in dogs, it can generate endocarditis and other severe pathology in humans, especially the immunosuppressed [35,[62], [63], [64]]. The *Bartonella* spp. taxon identified in one dog was most closely related to both *B. schoenbuchensis* and *B. chomelii*. The former pathogen is known to infect mammalian hosts such as deer and bison and is believed to be responsible for deer ked dermatitis in humans, whilst the latter pathogen is associated with infections of cattle [[65], [66], [67], [68], [69]]. A lower level of classification for this *Bartonella* taxon was not possible as for many species in this genus, even the full-length 16S rRNA gene does not provide sufficient discriminatory power to confidently classify species [70].

Phylogenetic placement (Fig. 3) confirmed the identity of *Dirofilaria* sp. hongkongensis from one Bhutanese dog. This filarial worm uses dogs as a reservoir host and although it is not known to generate any significant pathology in this species, it is zoonotic, and capable of causing complex infections in people [[11], [12], [13]]. For example, human *Dirofilaria* sp. hongkongensis infection has been associated with painful subcutaneous nodules that commonly affect sites around the eyelid and conjunctiva generating ocular complications, whilst surgical removal of adult worms places patients at risk of severe anaphylactic reactions [11,12,49,71,72]. More recently, this filarioid pathogen has also been found presenting as a breast tumour in a human patient [48]. *Dirofilaria* sp. hongkongensis has previously been identified in canines from southern India and Sri Lanka whilst human infections have been found further afield in China and Thailand as well as in European and Australian travellers with a history of travel to India and Sri Lanka [[11], [12], [13],^48^,^49^,[72], [73], [74], [75]]. Our identification of a range extension for this pathogen into Bhutan is therefore not surprising, given the prevalence of this pathogen in India [11] and that the sampling location from where the *Dirofilaria* sp. hongkongensis positive canine was detected is on the Bhutanese-Indian border.

The identification and confirmation through phylogenetic analyses (Fig. 4) of *S. tundra* in one Bhutanese dog is unusual given that this species is a known pathogen of cervids, such as reindeer (*Rangifer tarandus tarandus*) and roe deer (*Capreolus capreolus*) and has not been identified from a canine before [[76], [77], [78]]. *Setaria tundra* is transmitted by mosquitoes of the genus *Aedes,* and to a lesser extent *Anopheles,* and have been found parasitising cervids in temperate-to-cold regions of northern Europe, including Finland, Germany, Hungary, Poland and Slovakia [[76], [77], [78], [79], [80], [81]]. Therefore, the detection of this filarial worm species in the comparatively warm (12 °C – 31 °C) and wet region of Sarpang in southern Bhutan is particularly surprising. Identification of *S. tundra* in this dog may simply represent a transient or non-patent infection through blood feeding by a mosquito vector on a non-viable host species, i.e., a canine. Therefore, further investigation into whether this filarial worm species can produce patent infections in dogs would be of great value. In the context of this study, the possibility of the *S. tundra* detection being a false positive caused by cross-contamination with a *S. tundra* positive control available within our laboratory is extremely unlikely. This is because metabarcoding of positive controls was conducted in a different workspace and at a different time to the sequencing of Bhutanese samples with thorough sterilisation procedures consistently used between experiments. Additionally, the flow cell used for Bhutanese sample sequencing had not previously been for any other filarial worm metabarcoding and the *S. tundra* sequences obtained from the Bhutanese canine were found to have four single nucleotide polymorphisms (SNPs) when compared to the COI gene sequence obtained from our available *S. tundra* positive control from a mosquito [34].

This study also identified many common canine VBPs that are typically found in temperate to tropical environments, including the bacterial pathogens *A. platys, E. canis* and *M. haemocanis,* as well as the apicomplexans *B. gibsoni, B. vogeli* and *H. canis* [4,31,[82], [83], [84]]. Many of these VBPs are transmitted by *Rhipicephalus linnaei* ticks, with pathogen species such as *E. canis* causing severe and potentially fatal infections in canines, whilst VBPs such as *B. vogeli* and *A. platys* typically only impact the health of puppies, or adults in the context of coinfections [[85], [86], [87], [88]]. In addition, some of these pathogens may be transmitted non-vectorially, for example the potentially fatal piroplasmid *B. gibsoni* can be transmitted through fighting and aggressive interactions, whilst the transmission of canine haemotropic mycoplasmas is a debated field of research with strong evidence to suggest that these pathogens can be transmitted in the absence of arthropod vectors [83,[89], [90], [91]]. The identification of these canine VBPs in Bhutan is not surprising given that *A. platys*, *Babesia* spp., *E. canis*, and *H. canis* have been identified previously in northeastern India within regions directly bordering the south of Bhutan where our study was conducted [82]. Overall, the high percentages of VBP infected dogs we identified across the south of Bhutan highlights an important deficit in canine VBP treatment and control within the country, with the use of topical ectoparasiticides on dogs strongly recommended to assist in reducing local canine VBP transmission [92,93].

Coinfections with two or more VBPs were frequently detected, with the most common being a dual infection with *B. gibsoni* and *H. canis* (27%), followed by a triple infection comprising *B. gibsoni*, *H. canis* and *M. haemocanis* (6%), some dogs even had coinfections with four different VBPs (3%). Accurate detection of VBP coinfections is important given that they are associated with exacerbated disease pathology and more severe clinical signs when compared to mono-infections [17,94]. The high numbers of coinfections detected using our metabarcoding approach also highlights a key strength of these methods in their ability to detect all VBPs from a target group of interest accurately and comprehensively, in a manner that may be difficult-to-impossible to achieve using traditional molecular approaches [31,95].

Through this study's multivariable regression models canine age, sex and the presence of ectoparasites were identified as being significant predictors for canine VBP positivity (Table 4). The finding that juvenile dogs had a decreased odds of being VBP positive is unsurprising given that many stray, owned and community dogs may never receive treatment for VBPs and therefore the chances of acquiring one of these typically lifelong infections increases with age [83,96,97]. The finding that male dogs had an increased odds of being VBP positive may be being driven by pathogens such as *B. gibsoni* that can also be acquired through blood exchange during fighting, in addition to vectorial transmission [83,89]. The high levels of *B. gibsoni* infection across all study sites (45%) in combination with increased aggressive interactions between male dogs may be behind this finding [98,99]. Nonetheless, the small number of variables, particularly clinical signs, that were found to be predictive for VBP positivity is likely due to the very limited size of our dataset for which complete metadata was available (74 dogs total). In addition, the very high numbers of VBP positive dogs found across our study cohort (78%) also limits the strength of our model's ability to identify significant predictors of VBP positivity. Importantly, the potential presence of other pathogens, e.g. gastrointestinal nematode parasites, that were not investigated within the present study could be further impacting our model in a way that is unidentifiable with the data presently accrued. For the improved identification of predictors of VBP positivity within Bhutanese dogs in the future, a larger and more complete dataset should be obtained that also factors in the possible impacts of pathogens that do not reside in the blood.

Through the 16S rRNA gene-targeting metabarcoding method a large diversity of bacterial species were detected from the blood of the Bhutanese dogs sampled, including many that are not vectorially transmitted and have an unknown or putative pathogenic status (Table 5). Of note are the detection of species such as zoonotic *Helicobacter canis* in 1% of our study cohort and *Brucella pseudogrignonensis* in 2% of dogs [100,101]. The former pathogen can cause a recurrent fever and gastrointestinal symptoms in humans and is associated with pet dog contact [101,102], whilst the latter is recognised as an emerging pathogen of both immunodeficient and immunocompetent patients [100]. *Achromobacter xylosoxidans* infections were also found in 2% of the Bhutanese dogs sampled. This bacterial species can cause infectious endocarditis and pericarditis in canines, particularly in immunosuppressed individuals [[103], [104], [105]], whilst in humans *A. xylosoxidans* is an emerging and opportunistic hospital-acquired pathogen [[106], [107], [108]]. In addition, 2% of dogs were found to have *Kurthia gibsoni* present in their blood, this bacterial species has not been associated with pathology in canines before, but is recognised as a rare sexually transmitted zoonotic pathogen of humans [109,110]. The absence of *Rickettsia* spp. detection from the canines sampled is interesting given that a serological survey from 2015 identified high levels of exposure to SFG and TG *Rickettsia* in Bhutanese dogs [27]. Nonetheless, this difference in findings could be due to the transient nature of rickettsaemia in dogs making molecular detection unlikely [111].

Relative to other metabarcoding methods that use different deep-sequencing platforms our approach using three different assays on ONT’ MinION™ device is relatively cost-effective and rapid, with sufficient data accrued to comprehensively characterise all bacterial, apicomplexan, and filarial worm pathogens [[32], [33], [34]]. Due to the multiplexing capabilities of 96 samples or more on ONT’ ligation sequencing kits and the ability to wash and re-use flow cells we were able to process all four batches of samples on just two flow cells, bringing per sample analyses costs down to just AU$32 (excluding DNA extraction costs) for all three pathogen groups tested. To mitigate the risk of DNA carry over generating cross-contamination between sequencing batches [32] the same pathogen targeting assay was never run for a second time on the same flow cell. Runtimes were typically shorter than those used on Illumina sequencing equipment, and these could have been reduced further if flow cells had not been re-used [112]. Nonetheless, our metabarcoding approach did have some limitations including a lengthy NGS library preparation protocol involving two separate PCRs with respective bead clean-up steps [33,34]. Future modifications to these assays could make use of ONT’ native barcoding kits, e.g., SQK-NBD114.24 which only require one PCR step, alongside employment of the latest V14 chemistry to improve raw read quality and potentially reduce bioinformatic processing steps required to achieve a high-level of taxonomic classification accuracy. Forthcoming research will work to further analyse the samples collected through this study including the exploration of gastrointestinal parasites detectable from faecal samples and VBPs from ectoparasites collected from canines.

## 5 Conclusions

This study represents the first exploration of VBP diversity in Bhutanese dogs using molecular diagnostic methods. Given the almost total lack of data on canine VBP within Bhutan the use of deep-sequencing metabarcoding was an ideal method with which to comprehensively characterise bacterial, apicomplexan, and filarial worm pathogen diversity. The detection of ten well-recognised canine blood-borne pathogens (*A. reconditum, A. platys, B. gibsoni, B. vogeli, B. clarridgeae, Dirofilaria* sp. hongkongensis, *E. canis, H. canis, M. haemocanis* and *S. pseudintermedius*) and three unusual pathogens (*Bartonella* spp., an *Ehrlichia chaffeensis*-like bacterium and *S. tundra*) demonstrates the strengths of metabarcoding to elucidate diverse and complex pathogen communities, whilst also detecting rare and/or unexpected organisms that may have never been previously found in a given host species before [31]. Moreover, our identification of the presence of several zoonotic canine VBPs is critically important data with which to alert local clinicians to the risk of infections transmitted from dogs to humans, including the novel identification of *Dirofilaria* sp. hongkongensis, and potentially novel *Ehrlichia* and *Bartonella* species in Bhutan. The utilised metabarcoding approaches are explorative and do not rely on prior epidemiological information. The accrual of such data is important as it can aid in the selection of more economical and high-throughput diagnostic methods with which to screen larger animal cohorts to improve our understanding of VBP in the future and implement One Health centred approaches to local treatment and control programs in Bhutan [[29], [30], [31]].

## Ethics approval and consent to participate

Field work in Bhutan was conducted under the Livestock Technical Advisory Committee, Department of Livestock, Bhutan ethics permit: DOL/GEN-23/2020-2021/2202 granted 21.05.2021. Samples were collected from animals only after informed consent had been obtained by the relevant owner.

## Funding

This study was funded by the 10.13039/501100000923Australian Research Council (ARC) Future Fellowships scheme - Grant ID: FT200100732.

## CRediT authorship contribution statement

**Lucas G. Huggins:** Writing – review & editing, Writing – original draft, Visualization, Validation, Resources, Project administration, Methodology, Investigation, Formal analysis, Data curation, Conceptualization. **Ugyen Namgyel:** Writing – review & editing, Visualization, Resources, Investigation, Formal analysis, Data curation. **Pelden Wangchuk:** Writing – review & editing, Resources, Methodology. **Ushani Atapattu:** Writing – review & editing, Investigation. **Rebecca Traub:** Resources, Project administration, Conceptualization. **Vito Colella:** Writing – review & editing, Validation, Supervision, Resources, Project administration, Methodology, Investigation, Formal analysis.

## Declaration of competing interest

The authors declare that they have no known competing financial interests or personal relationships that could have appeared to influence the work reported in this paper.

## Data Availability

All nanopore sequencing data produced in this study are available from the NCBI BioProject database BioProjectID: PRJNA1066489.
